# Study on the Frying Performance Evaluation of Refined Soybean Oil after PLC Enzymatic Degumming

**DOI:** 10.3390/foods13020275

**Published:** 2024-01-16

**Authors:** Wenting Zhou, Yuxin Peng, Zongyuan Wu, Weinong Zhang, Yanxia Cong

**Affiliations:** 1 School of Food Science and Engineering, Wuhan Polytechnic University, Wuhan 430023, China; 18700600466@163.com (W.Z.); 18186455914@163.com (Y.P.); wzytotti@whpu.edu.cn (Z.W.); zhangweinong@163.com (W.Z.); 2 Key Laboratory for Deep Processing of Major Grain and Oil, Ministry of Education, Wuhan Polytechnic University, Wuhan 430023, China; 3 Hubei Key Laboratory for Processing and Transformation of Agricultural Products, Wuhan Polytechnic University, Wuhan 430023, China

**Keywords:** frying, soybean oil, degumming, diacylglycerol

## Abstract

It is known that phospholipase C (PLC) enzymatic degumming can hydrolyze phospholipids into diacylglycerol (DAG), which improves the efficiency of oil processing. However, it is unclear whether the presence of DAG and the use of enzymes affect the performance of the oil. This paper evaluated the frying performance of PLC-degummed refined soybean oil. Following the chicken wings and potato chips frying trials, results revealed that after 30 cycles of frying, free fatty acid (FFA) levels were 0.22% and 0.21%, with total polar compounds (TPC) at 23.75% and 24.00%, and peroxide value (PV) levels were 5.90 meq/kg and 6.45 meq/kg, respectively. Overall, PLC-degummed refined soybean oil showed almost the same frying properties as traditional water-degummed refined oil in terms of FFA, PV, TPC, polymer content, viscosity, color, foaming of frying oils, and appearance of foods. Moreover, FFA, TPC, polymer content, foaming, and color showed significant positive correlations with each other (*p* < 0.05) in soybean oil intermittent frying processing.

## 1. Introduction

Enzymatic degumming has been a topic of research and development for several decades since the launch of the first enzymatic degumming process, EnzyMax, in 1992 [1]. This process involves the use of phospholipases to remove gums from vegetable oils, such as soybean oil, thus improving their quality and yield. Initially, porcine phospholipase A2 derived from cobras was used as a phospholipase, but it was expensive and non-Kosher. Therefore, microbial phospholipases, such as Lecitase 10 L and LysoMax, were developed and widely used [1].

Phospholipases can be classified into different types based on their action sites on phospholipids. For example, phospholipase A1 (PLA1) catalyzes the hydrolysis of ester bonds at the 1-position of glycerol phospholipids, while PLA2 acts on ester bonds at the 2-position of phospholipids. Both PLA1 and PLA2 produce free fatty acids and lysophospholipids as hydrolysates [2]. On the other hand, phospholipase C (PLC) catalyzes the hydrolysis of the bond between the phosphate group and the glycerol moiety, resulting in the production of 1,2-diacylglycerol (1,2-DAG) and phosphate [3]. The utilization of PLC degumming in crude soybean oil enhances the oil yield due to the presence of 1,2-DAG, which remains in the oil. Soybean crude oil typically contains approximately 2–5% phospholipids, predominantly including PC, PE, PI, and PA. The presence of DAG in PLC-degummed soybean oil depends on the content and type of phospholipids in the crude oil [3,4].

The presence of 3-monochloropropane-1,2-diol (3-MCPD) in edible oil is primarily associated with the refining process. Through degumming, neutralization, and bleaching processes, the content of 3-MCPD can be reduced by 84%, 81%, and 84%, respectively [5]. Heat-induced reactions during deodorization, in particular, contribute to the formation of 3-MCPD and glycidyl esters [6]. During the refining process, partial acylglycerols, such as DAG and monoglyceride, have been identified as precursors to 3-MCPD formation. A study by Yao et al. indicated that 4% DAG was a critical level for 3-MCPD formation [7]. When comparing refined soybean oil and palm oil, DAG content is typically 1–3% in soybean oil, whereas palm oil exhibits higher levels ranging from 6% to 10%. Consequently, palm oil is more prone to form 3-MCPD [8].

Frying is a popular cooking technique used to produce a variety of fried foods, including potato chips, dough, and chicken wings, which are known for their golden color, good flavor, and crispy texture. During the frying process, heating and mass transfer occur, and the high temperatures of at least 150 °C, along with exposure to air and the presence of moisture in the food, lead to intensive hydrolysis, oxidation, polymerization, and other reactions in the oil [9,10]. These reactions cause changes in the physical properties and chemical composition of the oil and affect the quality of the fried foods. Physical parameters, including color and viscosity, as well as chemical parameters, such as free fatty acid (FFA) and total polar compounds (TPC), play a crucial role in evaluating the quality of frying oils. TPC, in particular, is considered an essential factor in determining frying oil quality [11,12]. According to the Chinese Vegetable Oil Standard (GB 2716-2018) [13], the acceptable TPC level for high-quality frying oils should not exceed 27% (*w*/*w*).

Soybean oil is the most widely used oil in cooking, frying, and other applications, owing to its reasonable price. As is known, one advantage of PLC-degummed refining oil is the higher oil yield than traditional degummed soybean oil due to the presence of DAG. Kasamatsu et al. found that DAG oil, both heated and unheated, did not show any genotoxic effect. Therefore, it can be concluded that DAG oil is safe to use under normal conditions [14]. Liu et al. reported foods fried in a high purity soybean derived DAG oil (SDO, >98.07%) had a more desirable texture and no harmful substances were detected [15]. Therefore, SDO could be a potential alternative to soybean oil for deep frying in the food industry, given its unique nutritional and pharmaceutical values.

However, compared to triacylglycerol (TAG), DAG has a lower boiling point and emulsifying properties, which make it different from TAG during the frying processes. Li et al. found that DAG is more susceptible to forming free fatty acids under frying conditions compared to TAG, and the smoke point of unheated DAG was approximately 40 °C lower than that of TAG [16]. Additionally, when subjected to frying for 3 h, the smoke point of the DAG declined more significantly than that of the TAG. Moreover, the emulsifying functionality of DAG may cause the oil to appear hazy when present in high quantities and increase the risk of excessive foaming during the frying process.

Numerous studies have reported on the development of phospholipase and optimization of enzymatic degummed processes, with less emphasis on the study of enzyme-treated oil stability. Thangaraju et al. found that the stability of degummed rapeseed oil was slightly lower than that of crude oil, which could be attributed to the antioxidant behavior of phospholipids [17]. Nosenko et al. reported that water-degummed sunflower oil and enzymatically-degummed oil had a similar antioxidative capacity [18]. They focused more on the stability of degummed oil, but in practice, the consumption form of soybean oil is refined soybean oil. However, there are limited data on enzymatically-degummed refined oil accumulation. It is unclear whether the use of enzymes would affect the stability of the refined oil. The purpose of this paper is to evaluate the frying performance of PLC enzymatically-degummed refined soybean oil and compare it with traditionally degummed refined soybean oil.

## 2. Materials and Methods

### 2.1. Materials

Chicken wings and potato chips were purchased from a local supermarket. Fresh soybean oil (with no antioxidants added) was provided by Yi Hai Kerry Co., Ltd (Shanghai, China). Potassium hydroxide, phenolphthalein, potassium iodide, sodium thiosulfate, isopropyl alcohol, ethyl ether, ethanol, chloroform, and glacial acetic acid were purchased from Sinopharm Group Co., Ltd. (Beijing, China).

### 2.2. Sample Preparation

Water-degummed refined soybean oil (WD-L) and PLC-degummed refined soybean oil (ED-L) were prepared in the laboratory. Using the same batch of raw soybean oil as the material, both traditional degumming (using a combination of water degumming and acid chelation) and PLC degumming were employed to obtain degummed oil. The traditional degummed oil was further processed through neutralization, bleaching, and deodorization to obtain refined oil. However, PLC-degummed refined oil is produced by directly subjecting enzymatically-degummed oil to bleaching and deodorization, bypassing the neutralization step. The enzymatic degumming process adopted in the laboratory was in accordance with reference procedures [3]. The process parameters for each individual process are depicted in Table S1.

### 2.3. Frying Procedure

Three different types of frying oil were tested in the processing of chicken wings and potato chips. These oils included factory-refined soybean oil (WD-P), WD-L, and ED-L. Each group underwent 30 frying cycles with the temperature of the oil set to 210 °C for four chicken wings (about 200 g, fried for 6 min) and 180 °C for 200 g potato chips (fried for 4 min), using 2.5 kg oil in a deep fryer. Ten consecutive batches were fried each day, and after every ten cycles, the oil was burned in the air for 2 h, then cooled naturally and stored at room temperature to simulate intermittent operation in a restaurant setting. The oil was not replenished or changed throughout the process, and the oil temperature remained at either 180 °C or 210 °C. Oil samples were collected from the 5th, 10th, 15th, 20th, 25th, and 30th batches and stored in a refrigerator until analysis.

### 2.4. Analysis of Oil Properties

#### 2.4.1. Content of TPC, FFA, Peroxide Value (PV), Color, and Foaming

TPC content was determined using an automatic rapid tester of Testo270 [19]. A total of 50 g fried oil was taken out of the deep-frying pot and cooled down to a temperature of 40 °C before conducting measurements. FFA results were expressed in % (as oleic acid) and were determined by AOCS Official Method Ca 5a-40. PV was expressed in meq/kg, determined following AOCS Official Method Cd 8b-90. Color was determined using AOCS Official Method Cc 13e-92 with the Lovibond Tintometer color scale and expressed as red and yellow values. The foam height was determined by measuring the increase in liquid level using a measuring scale during the frying process.

#### 2.4.2. Polymer Content

The polymer content was determined following the method described by Khor [20]. Firstly, the nonpolar fractions were separated by washing with a hexane-diethyl ether mixture (90:10, *v*/*v*). Subsequently, the polar fractions were collected by washing with diethyl ether. The polar fraction was then dissolved in tetrahydrofuran and subjected to analysis using a Shimadzu HPLC system, which included an SIL-10AD injector, LC-20AD pump, and RID-10A refractive index detector. The polar fraction was separated into two size exclusion columns in series (Phenogel, 100 Å, 100 Å; 7.8 mm × 300 cm, internal diameter, Phenomenex, Torrance, CA, USA), and the column temperature is 35 °C. The mobile phase used for separation was tetrahydrofuran at a flow rate of 1 mL/min. The sum of oxidized triglyceride oligomers, oxidized triglyceride dimers, and oxidized triglyceride monomers will be referred to as polymers.

#### 2.4.3. Viscosity

The viscosity of the oils was determined using a Brookfield DV2T viscometer (Brookfield Engineering Laboratories Inc., Middleboro, MA, USA) [21]. The procedure involved applying 1 mL of the oil sample onto the viscometer plate, which was equipped with a spindle RV-04. The viscosity value (kinematic viscosity, mPa s) was then directly read from the viscometer, which was kept at a constant temperature of 25 °C.

### 2.5. Statistical Analysis

The experiments were carried out in triplicate, and the data have been presented as mean values along with the standard deviation. The statistical analysis of the collected data was conducted using IBM’s statistical software (SPSS^®^, version 20, IBM Corp., New York, NY, USA). One-way analysis of variance (ANOVA) and correlation analysis were employed to analyze the data. Duncan’s Multiple Range Test was chosen to locate the differences between means, and a significance level of 5% (*p* < 0.05) was considered acceptable.

## 3. Results and Discussion

Fried foods can be broadly categorized into high-protein starch and high-starch foods, with fried chicken wings and legs representing the former while French fries and potato chips represent the latter. To accurately evaluate the frying properties of degummed refined oil, both traditionally degummed and PLC-degummed refined soybean oils were selected. To account for any potential differences in frying performance due to equipment or laboratory settings, we also conducted frying experiments using WD-P, thereby ensuring more reliable results. It is worth noting that all refined oils utilized in the study were free from any added antioxidants.

### 3.1. Free Fatty Acid Value

The frying process involves the contact between oil, air, moisture, and materials at higher temperatures, which can cause changes in the quality of the oil. The presence of moisture can prompt the hydrolysis of triglycerides, leading to the formation of diglycerides, monoglycerides, free fatty acids, and other by-products, thereby increasing the free fatty acid content. Furthermore, high temperatures can also intensify the hydrolysis reaction.

**Figure 1:**
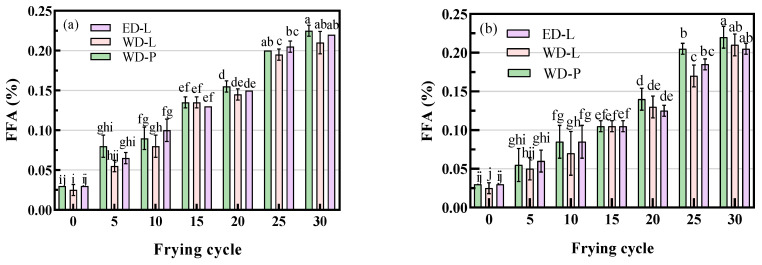
FFA of chicken wings and potato chips frying oils, (**a**) chicken wings; (**b**) potato chips, different small letters in the same items indicate a significant result as determined by Duncan’s range test (*p* < 0.05), according to the treatment method.

FFA content is a crucial parameter in assessing oil quality. According to the Chinese Vegetable Oil Standard (GB 2716-2018), first-grade refined vegetable oil should have an FFA content below 1.5%, while frying oil should not exceed 2.5% of free fatty acids. Figure 1a illustrates the variation in FFA content in chicken wings frying oil after different frying cycles. The initial FFA content of fresh oils was consistently below 0.05%, and there were no significant differences (*p* < 0.05) among them. With the increase in temperature, the FFA content rose in all frying oils. This gradual increase aligned with findings in the existing literature [20,21]. After 30 frying cycles, the FFA content for WD-P, WD-L, and ED-L was 0.23%, 0.22%, and 0.22%, respectively. Moreover, there were no significant differences (*p* < 0.05) in the increase in FFA among the three types of frying oil.

In the potato chips frying experiment, FFA content showed a gradual increase with frying cycles, and there were no significant differences (*p* < 0.05) observed among the different frying oils. After 30 frying cycles, the FFA content (Figure 1b) for WD-P, WD-L, and ED-L was 0.22%, 0.21%, and 0.21%, respectively. Compared to the frying of chicken wings, the increase in FFA was slightly lower. This could be attributed to the higher moisture content in potato chips (65.6%) compared to chicken wings (56.8%), which promotes hydrolysis [22]. Moreover, the longer frying duration and higher temperature of chicken wings, as well as the presence of rich unsaturated fatty acids and metal ions in chicken wings, may contribute to the difference in FFA levels. After every 10 cycles of frying, the oils were maintained without heating foods (to simulate intermittent operation in fast food restaurants) for 2 h, then cooled overnight. After five cycles of frying, the FFA in frying oil increased by approximately 0.01~0.03% (chicken wings, 5th to 10th cycle). However, FFA content increased by approximately 0.03~0.06% (chicken wings, 10th to 15th cycle), which was obviously higher. This clearly demonstrated that during intermittent frying, in addition to the degradation of oil caused by frying food, the continuous heating also contributes to the deterioration of the oil, and the phenomenon of dry heating of an empty pan should be minimized as much as possible in intermittent operation. Overall, the increase in FFA during frying was slow, and after 30 frying cycles, the FFA content remained well below the relevant standards for frying oil.

The impact of DAG on the formation of FFA during the frying process has been debated in various articles. Li et al. suggested that DAG is less stable than TAG and susceptible to the formation of free fatty acids during frying [16]. However, Liu et al. found no significant differences in variations of FFA between SBO and SDO during the frying [15]. Our results showed that ED-L with a 1.89% DAG content did not have any significant differences compared to traditionally degummed refined soybean oil [10].

### 3.2. Peroxide Value

The oxidation of oils can diminish the quality and nutrition while also imparting unpleasant flavors and odors. Oxygen, high temperatures, moisture, and metal ions are the primary factors that cause oil oxidation. PV is a commonly used indicator for assessing the degree of oxidation. During the initial stages of oil oxidation, the formation of unstable peroxides initiates complex reactions, leading to the generation of secondary compounds, including aldehydes, ketones, acids, alcohols, epoxides, or polymerized products. These compounds contribute to the emergence of strong and unpleasant odors. Furthermore, oxidation can result in the degradation of pigments, flavor compounds, and vitamins, thereby intensifying the process of rancidity [10].

**Figure 2:**
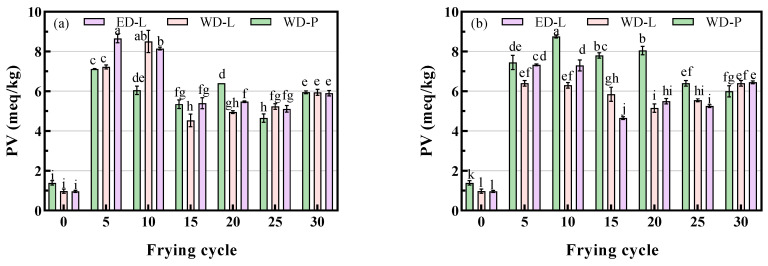
PV of chicken wings and potato chips frying oils, (**a**) chicken wings; (**b**) potato chips, different small letters in the same items indicate a significant result as determined by Duncan’s range test (*p* < 0.05), according to the treatment method.

The PV levels of fresh oil were 1.39 meq/kg, 0.98 meq/kg, and 0.96 meq/kg, respectively, which were all below the threshold of 19.7 meq/kg (Chinese Edible Oil Standard, GB 2716-2018). Figure 2 illustrates the changes in PV during frying. Regardless of whether they were chicken wings or potato chips, the PV exhibited an initial increase, followed by a decrease, then an increase again. The observed trend in PV corresponded with previous findings by Choe et al., which indicated the simultaneous production and decomposition of peroxides during oxidation [10]. Initially, the PV of frying oil from chicken wings was significantly higher than that of frying oil from potato chips. However, after 15 frying cycles, the PV decreased markedly and eventually fell below that of the frying oil from potato chips. This could be attributed to the longer frying duration and higher temperatures used for frying chicken wings, which led to increased peroxide decomposition and the generation of smaller molecules. Hydrolysis may have also played a role, resulting in the formation of more free fatty acids, as evidenced by the slightly higher FFA content in the fried chicken wings compared with the potato chips. Notably, no significant differences were observed between ED-L and WD-L/WD-P, which is consistent with findings reported by Liu et al. [15,23].

### 3.3. Total Polar Compounds

During the frying process, the repeated use of frying oil at high temperatures can trigger reactions such as oxidation, polymerization, cracking, and hydrolysis. These reactions produce polar compounds, including carbonyls, carboxyls, ketones, and aldehydes, which possess higher polarity compared with triglycerides. The total polar compound content indicated the presence of polar molecules, and the Chinese Edible Oil Standard GB 2716-2018 sets a maximum limit of 27% for polar compound content in frying oil. Research has shown that polar compounds could stimulate inflammatory responses in the body, and TPC shows a significant correlation with the levels of diacylglycerols (DAG) and FFA [22,24].

Fresh fried oil naturally contains some polar components, including monoglycerides, tocopherols, and plant sterols, which means its polar compound content is not zero. PLC hydrolyzes the phospholipids bond to produce DAG and phosphoric acid esters. The content of DAG in ED-L is 1.86%, while in the WD-P and WD-L is 0.27% and 0.31%, respectively. As is known, ED-L and WD-L have adopted different refined methods, which resulted in differences between them in the content of tocopherols and phytosterols. Finally, the TPC of WD-P and WD-L is 7.75%, while in ED-L, it is 8.50%, slightly higher than that of traditional processes. The initial TPC levels in the fresh oil mentioned above are consistent with those reported by Khor [20].

**Figure 3:**
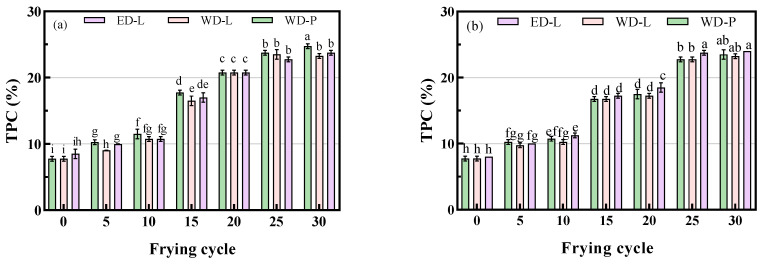
TPC in chicken wings and potato chips frying oils, (**a**) chicken wings; (**b**) potato chips, different small letters in the same items indicate a significant result as determined by Duncan’s range test (*p* < 0.05), according to the treatment method.

The increasing trend of TPC in frying oil is shown in Figure 3. With an increase in the number of frying cycles and the duration of oil usage, TPC exhibited a linear increase, which is consistent with the findings of Aladedunye et al. [11]. After 30 frying cycles, the TPC levels were below the national standard limit of 27%. The TPC in fried oil from chicken wings (Figure 3a) increased from 7.75%, 7.75%, and 8.50% to 24.75%, 23.25%, and 23.75%, respectively, while that in potato chips (Figure 3b) increased to 23.50%, 23.25%, and 24.00%. The difference in TPC among oils was not significant (*p* < 0.05). Overall, the TPC increased slightly faster in fried chicken wings than in potato chips due to the higher temperature and longer frying time. Chicken wings contain approximately 9.00 g of unsaturated fatty acids per 100 g, whereas the fatty acid content in potato chips (3.99%) is much lower [22]. Despite having a slightly higher initial TPC content, ED-L did not exhibit a significant difference in the rate and extent of TPC increase during frying compared to traditionally refined oils.

### 3.4. Polymer Content

The polymer content in fresh soybean oil was relatively consistent, approximately around 3.30%. This can be attributed to the high temperature and moisture in the refining process, which leads to the formation of polymers. During the frying process, glycerides and fatty acids undergo oxidation and thermal polymerization, resulting in the formation of larger molecules, such as oxidized glyceryl trimers, oxidized glyceryl dimers, oxidized glyceryl monomers, and cyclic fatty acid monomers [20,22,25].

**Figure 4:**
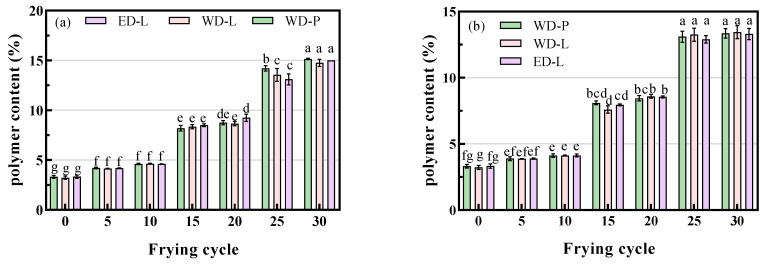
Polymer content of chicken wings and potato chips frying oils, (**a**) chicken wings; (**b**) potato chips, different small letters in the same items indicate a significant result as determined by Duncan’s range test (*p* < 0.05), according to the treatment method.

The polymer content in the oil during the frying process is shown in Figure 4. As frying time increased, polymer content in the oil increased, which led to higher levels of these compounds. In ED-L, polymer content increased from 3.34% to 15.00% (chicken wings frying, Figure 4a) and 13.30% (potato chips frying). After 5, 10, 15, 20, 25, and 30 frying cycles, the polymer contents in ED-L (chicken wings, Figure 4a) were 3.34%, 4.19%, 4.61%, 8.50%, 9.25%, 13.10%, and 15.00%, respectively, while those in potato chips (Figure 4b) were 3.89%, 4.14%, 7.95%, 8.55%, 12.90%, and 13.30%, respectively. During cycles 10–15 and 20–25, there was a significant increase in the polymer content of the frying oil, which can be attributed to the continuous heating of the oil for 2 h after every 10 frying cycles. Taking ED-L as an example, the polymer content increased by 0.42% (chicken wing, 5th to 10th) and 3.89% (chicken wing, 10th to 15th), which was consistent with the trends observed in TPC and FFA.

The rate of polymerization in frying oil from chicken wings was slightly higher than that of potato chips, which can be attributed to their different compositions. Potato chips consist mainly of starch, while chicken wings contain a high content of unsaturated fatty acids and proteins, both of which can promote the production of polymers. Hwang et al. revealed that ester bonds are responsible for soybean oil polymerization during frying and heating at 175 °C [21]. Overall, the rate of polymerization of ED-L during frying does not show a significant difference from that of traditionally refined oil.

### 3.5. Viscosity

The viscosity of oil depends on its constitution, particularly its fatty acid content, and distribution, as well as the presence of the other lipids. Frying involves the exposure of oils to moisture, high temperatures, and oxygen, which promote degradation, oxidation, and polymerization, leading to an increase in viscosity. Additionally, the migration of components within the fried food also contributes to the increase in viscosity. Changes in viscosity during frying were correlated with the levels of oleic acid and linoleic acid. Under the same frying conditions, soybean oil, with its high linoleic acid content, oxidizes more easily, resulting in a faster viscosity increase than palm oil [19]. Oil type and temperature had a significant effect on viscosity, and high temperatures promote molecular movement and weaken intermolecular interactions, resulting in decreased oil viscosity. As reported, the viscosity of fried oil was higher than that of fresh oil at room temperature [26].

**Figure 5:**
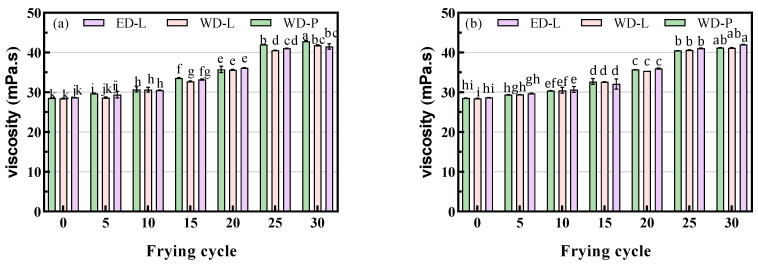
Viscosity of chicken wings and potato chips frying oils, (**a**) chicken wings; (**b**) potato chips, different small letters in the same items indicate a significant result as determined by Duncan’s range test (*p* < 0.05), according to the treatment method.

The viscosity (detected at room temperature) of fresh ED-L was found to be 28.67 mPa s, which is indistinguishable from that of traditionally refined oils (28.48 mPa s and 28.57 mPa s). After 5, 10, 15, 20, 25, and 30 frying cycles, the viscosities of ED-L (chicken wings, Figure 5a) were observed to be 29.91, 30.53, 33.22, 36.14, 41.08, and 42.03 mPa s, respectively. Concurrently, those for potato chips (Figure 5b) frying oils were 29.73, 31.20, 33.09, 35.98, 41.08, and 42.01 mPa s, respectively. After 30 cycles of frying, the viscosity increased by 46.60% for chicken wing frying oil and 46.50% for potato chips frying oil. The viscosity of oils increased obviously with each frying cycle, with no notable differences observed among those of different degumming processes. These viscosity changes are consistent with the trends observed in FFA, PV, TPC, and polymer content. Furthermore, the frying oil from chicken wings had a slightly higher viscosity than that of the potato chips’ frying oil. These variations in viscosity can be attributed to the specific frying conditions and the nature of the food.

### 3.6. Foaming and Color

The color of cooking oil is an important indicator for consumers, and it is influenced by nonenzymatic browning reactions, such as the Maillard and caramelization reactions. These reactions lead to the formation of brown, black, and golden caramel pigments in fried food. After decolorization and high-temperature refining, the color of the oil becomes very light (Table 1), with R of 1.00 (WD-P), 0.40 (WD-L), and 0.70 (ED-L), respectively. Due to differences in the refining process, such as alkaline and deodorization time, the initial colors of the three oils varied slightly, but they still met the relevant standards for edible oil.

**Figure 6:**
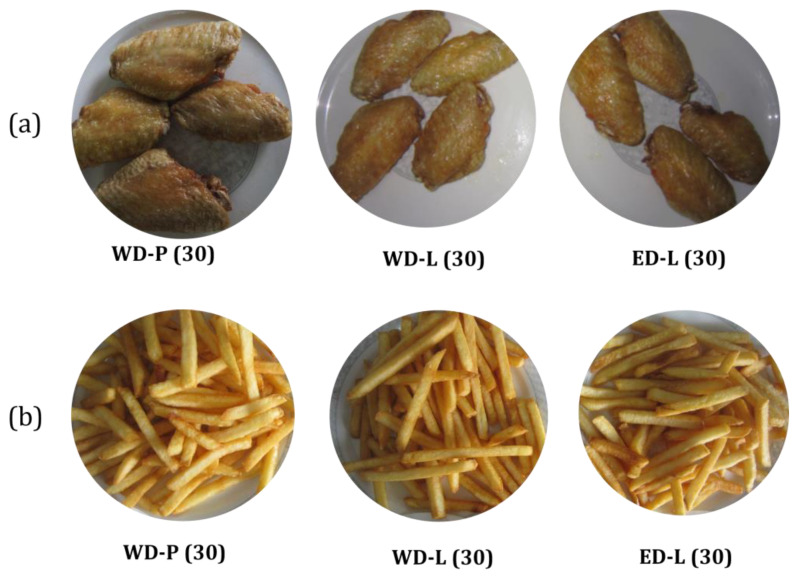
The appearance of the chicken wings and potato chips after 30 cycles of frying. (**a**) chicken wings; (**b**) potato chips.

The color of the oil changed significantly during the frying process. After 30 cycles, the frying oil appeared maroon, and the fried chicken wings showed a reddish-brown appearance (Figure 6). Following 30 frying cycles, the color of the oil intensifies, as shown in Table 1 by the values WD-P (R18.00), ED-L (R20.20), and WD-L (R22.00). Visually, the variations in the appearance of the fried foods (Figure 6) among these three were deemed acceptable. Overall, there were no substantial variations in color across different degumming techniques.

Frying oil can foam due to the presence of polymers, polar compounds, and water.

**Figure 7:**
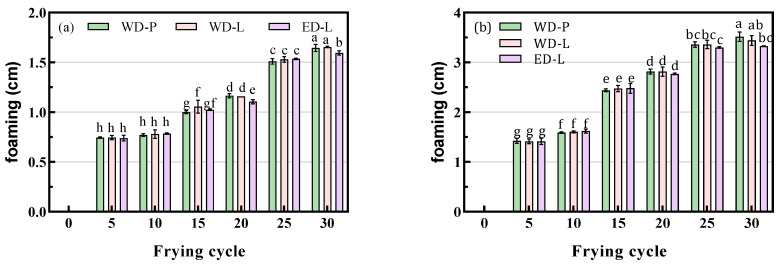
Foaming height of chicken wings and potato chips frying oils, (**a**) chicken wings; (**b**) potato chips, different small letters in the same items indicate a significant result as determined by Duncan’s range test (*p* < 0.05), according to the treatment method.

DAG, as an emulsifier, could promote foaming. Therefore, the foaming height of the oil after frying was measured to assess its foaming properties. From Figure 7, it can be observed potato chip frying exhibited more foaming compared with chicken wing frying, which could be attributed to the moisture content and weight of foods. After 30 cycles of frying, the foaming height of WD-P, WD-L, and ED-L were 1.65, 1.66, and 1.60 cm (Figure 7a), which were 3.52, 3.44, and 3.33 cm (potato chips, Figure 7b), respectively. Overall, there was no significant difference in foaming performance between ED-L and WD oils.

### 3.7. Correlation Analysis

In order to further explore the relationship among different frying evaluation indexes, a Pearson correlation analysis was conducted, and the results are shown in Table 2. The variables FFA, TPC, polymer content, foaming, and color showed a significant positive correlation with each other (*p* < 0.01). Aladedunye et al. reported a strong correlation observed between color and TPC [11]. Polymer content exhibited a significant positive correlation with TPC [22]. However, PV showed a different trend compared with the others. PV exhibited a significant positive correlation with color and FFA, but no correlation was found between PV and the other variables. The result was consistent with Khor et al., illustrating that PV may not be an appropriate parameter for evaluating the quality of frying oil due to its limitation in measuring only the primary oxidation products [20].

## 4. Conclusions

In the present work, we evaluated the frying performance of PLC-degummed refined soybean oil, focusing on FFA, PV, TPC, polymer content, color, and foaming value in chicken wing and potato chips trials. After 30 cycles of frying, FFA levels were 0.22% and 0.21%, with TPC at 23.75% and 24.00%, and PV was 5.90 meq/kg and 6.45 meq/kg, respectively. Overall, the enzymatic degummed refined oil exhibited almost the same frying properties as traditionally degummed refined oil. The DAG (accounted for 1.86%) in enzymatic degummed refined soybean oil did not significantly affect TPC, polymer content, and foaming height. Concurrently, there was no increase in FFA and PV due to the use of enzymes in degumming. Additionally, FFA, TPC, polymer contents, foaming, and color showed significant positive correlations with each other (*p* < 0.05) in the soybean oil frying process. These results will contribute to the promotion of enzymatic degummed technology in the edible oil processing industry.

## Figures and Tables

**Table 1:** Color of frying oil from chicken wings and potato chips under different frying cycles.

Food	Cycles	WD-P	WD-L	ED-L
Chicken wings	0	R1.0 Y10	R0.4 Y3.0	R0.7 Y6.0
	5	R2.5 Y40	R2.3 Y20	R2.3 Y20
	10	R4.5 Y50	R4.0 Y40	R4.0 Y30
	15	R7.0 Y60	R8.0 Y40	R6.5 Y40
	20	R9.4 Y21	R12.0 Y50	R10.3 Y40
	25	R14.3 Y60	R19.0 Y50	R16.3 Y30
	30	R18.0 Y70	R22.0 Y60	R20.2 Y60
Potato chips	5	R2.0 Y40	R2.0 Y40	R2.0 Y30
	10	R3.0 Y20	R2.5 Y30	R3.0 Y50
	15	R4.0 Y40	R6.0 Y50	R5.0 Y40
	20	R5.0 Y60	R6.0 Y60	R5.0 Y40
	25	R8.0 Y70	R11 Y70	R9.2 Y70
	30	R9.5 Y70	R11 Y70	R10 Y70

**Table 2:** Correlation coefficients (*r*) of frying parameters.

	PV	FFA	TPC	Polymers	Viscosity	Foaming	Color
PV	1						
FFA	0.297 **	1					
TPC	0.206	0.966 **	1				
polymers	0.151	0.965 **	0.973 **	1			
viscosity	0.162	0.849 **	0.880 **	0.882 **	1		
foaming	0.485 **	0.550 **	0.566 **	0.524 **	0.448 **	1	
color	0.142	0.887 **	0.870 **	0.903 **	0.808 **	0.306 **	1

** Highly significant differences at *p* < 0.001.

## Data Availability

Data is contained within the article or supplementary material.

## Supplementary Materials

The following supporting information can be downloaded at: https://www.mdpi.com/article/10.3390/foods13020275/s1.
