# Supplementary material for: Study on the Frying Performance Evaluation of Refined Soybean Oil after PLC Enzymatic Degumming
Source: Foods. 2024 Jan 16;13(2):275. doi: 10.3390/foods13020275 (PMC10815329; doi:10.3390/foods13020275)
Supplement: Supplementary file 1 [file foods-13-00275-s001.zip › foods-2810095-supplementary.pdf]

Table S1. Soybean oil refining process and main parameters

Process		Parametres	ED-L	WD-L
		Oil weight (g)	700	700
Water degumming		Water dosage (%)		3%
		Temperature (° C)		85
		Time（min）		20
PLC degumming	Citric acid addition	Citric acid monohydrate concentration (w/w)	50	
		Citric acid monohydrate solution dosage (g)	0.56	
		High shear mixer, 10,000 rpm time (min)	2	
		Overhead mixer time (min)	30	
	Cooling	Temperature (° C)	60	
	NaOH addition	NaOH solution (w/w, %)	16	
		NaOH solution dosage (g)	0.875	
		Agitation time (min)	30	
		Water dosage (g)	12.46	
		PLC dosage (g)	0.105	
		High shear mixer, 10,000 rpm time (min)	2	
	Enzyme reaction	Temperature (° C)	55	
		Time (min)	120	
	Deactivation	Temperature (° C)	85	
Centrifuge		Temperature (° C)	85	85
		Centrifuge strength (G)	4000	4000
		Centrifuge time（min）	15	15
Chelation		Oil temperature (° C)		85
		Phosphoric acid concentration (w/w, %)		85
		Phosphoric acid dosage (g)		0.133
		Agitation time (min)		10
Neutralization		NaOH solution concentration (w/w, %)		7.3
		NaOH solution dosage (g)		9.24
		High Shear Mixer, 10,000 rpm time (min)		10
Drying& Bleaching		Vacuum (mbar)	100	100
		Bleaching earth dosage (%)	1.5	1.5
		Active carbon dosage (%)	0.1	0.1
		Time (min)	30	30
		Temperature (° C)	105	105
Deodorization		Temperature (° C)	240-245	240-245
		Time (min)	120	120
		Vacuum (mbar)	5-10	5-10
